# The Effects of Hyaluronic Acid on Gait Parameters in Patients with Knee Osteoarthritis: A Systematic Literature Review

**DOI:** 10.3390/medicina61081488

**Published:** 2025-08-20

**Authors:** Cosimo Costantino, Sara Ronzoni, Annalisa Ingletto, Roberto Sabato, Antonello Salerno, Stefano Palermi, Ruben Foresti, Chiara Martini, Andrea Demeco

**Affiliations:** 1 Department of Medicine and Surgery, University of Parma, 43126 Parma, Italy; cosimo.costantino@unipr.it (C.C.); sara.ronzoni@unipr.it (S.R.); annalisa.ingletto@unipr.it (A.I.); roberto.sabato@unipr.it (R.S.); ruben.foresti@unipr.it (R.F.); chiara.martini@unipr.it (C.M.); 2 Department of Medicine and Surgery, UniCamillus Saint Camillus International University of Health Sciences, 00131 Rome, Italy; stefano.palermi@unicamillus.org; 3 Physical and Rehabilitative Medicine, Department of Medical and Surgical Sciences, University of Catanzaro “Magna Graecia”, 88100 Catanzaro, Italy

**Keywords:** gonarthritis, hyaluronic acid, gait analysis, inertial sensors, electromyographic sensors, rehabilitation

## Abstract

*Background and Objectives*: Knee Osteoarthritis affects about 10% of people over 50, causing pain and functional limitation. Hyaluronic acid (HA) is crucial in regulating the osteocartilaginous matrix. Patients are usually assessed using clinical scores to examine symptoms and quality of life, and in this context, gait analysis could provide an objective assessment of walking patterns to identify any deficits. This systematic review investigates the short and long-term effects of intra-articular HA injections on gait kinematics, pain and activities of daily living (ADL), investigating the correlation between outcomes. *Materials and Methods*: The review followed PRISMA guidelines. The PICO model included patients with radiographic knee osteoarthritis who received intra-articular HA injections, comparing them to healthy controls or those receiving corticosteroids or placebo. Outcomes included gait kinetics and functional scales at baseline and during follow-ups. *Results*: From 342 identified articles, 13 were included, comprising a total of 321 patients. The gait analysis utilized optoelectronic systems, inertial sensors, and electromyographic sensors pre- and post-HA treatment. Clinical parameters were assessed using the Visual Analogue Scale, WOMAC OA, Knee Society Score, Lequesne Score, and SF-36. The data showed significant improvement in speed (*p* = 0.001) and step cadence (*p* < 0.005) 30 days post-treatment and improvements in knee adduction moment (*p* < 0.001) and sagittal ground reaction force vectors (*p* < 0.01) up to six months post-treatment. Pain reduction and improvements in VAS (*p* < 0.001) and Lequesne score (*p* < 0.001) were observed in short-term follow-ups. *Conclusions*: Our study suggests an improvement in pain and knee function after hyaluronic acid injection. Moreover, gait analysis is an important tool for objectively assessing deficits and developing personalized rehabilitation programs. Furthermore, combining infiltrative treatment with rehabilitation could extend the effects of hyaluronic acid and improve results.

## 1. Introduction

Knee osteoarthritis (KOA) is among the most prevalent chronic health conditions resulting from progressive changes in the joint microenvironment and marked by pain and functional impairment. The prevalence of symptomatic knee OA varies across studies, although it is one of the most studied joints. Its incidence increases with age, and its onset is influenced by intrinsic, unmodifiable risk factors such as ethnicity, age, gender, and family history as well as extrinsic factors like obesity, overuse, and a history of previous traumas [1,2,3].

The diagnosis of KOA involves a combination of complementary clinical and radiographic signs [4]. Clinically, patients complain of persistent pain and worsening mobility, both nocturnal and during weight-bearing activities [4]. From a radiological point of view, KOA can be classified using the Kellgren–Lawrence scale. However, not all patients with radiological KOA diagnosis present symptoms and, conversely, patients in the early stages may experience pain despite a radiological examination yielding basically negative results [5].

Under examination, KOA presents with joint stiffness and reduced range of motion (ROM), local redness and warmth, swelling and synovial fluid effusion, crepitus, and the sensation of joint locking. Lastly, instability and decreased muscle tone in the affected limb may occur [6].

Clinical scales are useful in assessing motor limitations. Namely, the Visual Analog Scale (VAS), the Knee Society Scale (KSS), the Knee Injury and Osteoarthritis Outcome Score (KOOS), the Western Ontario and McMaster Universities Osteoarthritis Index (WOMAC), and the Algofunctional Index of LEQUESNE evaluate knee pain and function [7,8,9]. In addition, quality of life assessment scales and daily activity scales such as ADL (Activities of Daily Living), IADL (Instrumental Activities of Daily Living), and the Short Form Health Survey 36 (SF-36) are useful to evaluate the impact of KOA on daily life [10]. In this context, the study of gait patterns plays a fundamental role in the early identification of biomechanical alterations in KOA and in the assessment of the patient’s deficits before and after treatment [11]. It allows an objective evaluation of the alteration of the walking pattern, guiding clinicians in the development of personalized rehabilitation programs based on their patients’ needs [11,12]. Moreover, technological development has provided physicians with several tools, including inertial sensors that utilize accelerometers, magnetometers, and gyroscopes; surface electromyographic instruments for assessing muscle excitation and coordination; and tools enabling the kinematic analysis of gait through motion capture systems based on infrared camera [13,14].

Several therapeutic approaches have been proposed for the management of KOA [1,15], depending on the Kellgren–Lawrence radiographic classification and comorbidities. However, none of these approaches has demonstrated the ability to modify substantially the progression of the disease. The chronic advancement of the disease needs a systematic and step-by-step approach, starting from conservative to surgical approaches, such as total knee replacement (TKR) [16,17].

Among the conservative interventions, lifestyle modifications, orthoses and footwear, muscle strengthening, aerobic exercise programs, and intra-articular (IA) injections are the most common utilized [18,19,20,21]. Different studies have shown that the administration of intra-articular drugs provides significant benefits for KOA treatment in terms of symptoms and functionality [22,23,24]. A study by Ishijima et al. reports that IA therapy is more effective than oral non-steroidal anti-inflammatory drugs and other systemic pharmacological treatments [25]. In particular, corticosteroids, supplementation of the synovial liquid, platelet-rich plasma (PRP), and mesenchymal stem cells (MSC) are the most studied substances in the literature [4,6,22,23]. However, the most widely injected drug is hyaluronic acid (HA) [26,27,28], a viscoelastic mucopolysaccharide, aiming to restore the physiological synovial fluid of the joint with a well-established safety profile [21].

Chronic knee pain often leads to the emergence of compensatory gait patterns aimed at minimizing discomfort by reducing joint load. However, these adaptations may persist even after the pain subsides. For this reason, a comprehensive rehabilitation strategy is essential, combining hyaluronic acid (HA) injections with targeted physiotherapy protocols to address and improve altered gait patterns. In this context, the evaluation of gait parameters represents a valuable method for objectively assessing joint performance, neuromuscular adaptation, and overall rehabilitation progress [26,28]. However, there is still limited consensus regarding the efficacy of HA injections on gait biomechanics.

Thus, the primary aim of this systematic review is to evaluate both the clinical efficacy and the biomechanical impact of intra-articular hyaluronic acid injections on knee function, with a specific focus on gait parameters assessed through kinematic analysis.

## 2. Materials and Methods

The Preferred Reporting Items for Systematic Reviews and Meta-Analyses (PRISMA) guidelines were followed for screening the following databases: PubMed, Web of Science, and Scopus. All sources were last consulted in June 2025. A systematic search was conducted for articles in the English language following the strategy outlined in Table 1. PROSPERO registration: CRD42024553910.

### 2.1. Selection of Articles

The following PICO model was used:

- Patients: patients with knee osteoarthritis (Grade I-II-III according to Kellgren–Lawrence and Ahlback);
- Intervention: IA injections of HA;
- Comparison: Healthy controls or individuals receiving corticosteroid injections or placebo;
- Outcomes: measurements of gait kinetics and functional assessment scores at baseline, and after follow-up.

We included randomized controlled trials (RCTs), case–control studies, and feasibility studies in the study design, with the full text available and written in English, that met the following inclusion criteria: patients, both male and female, with radiographically diagnosed knee osteoarthritis who received intra-articular hyaluronic acid injections.

Only studies employing quantitative gait analysis for outcome assessment—specifically using optoelectronic motion capture systems, force platforms, or multi-sensor motion analysis technologies—were included.

Studies comparing intra-articular hyaluronic acid injections with other injection therapies—such as platelet-rich plasma (PRP) or corticosteroids—or with placebo were also considered. We also reviewed the references of the included studies to detect additional research that may have been missed.

The exclusion criteria eliminated studies involving additional treatments besides hyaluronic acid injections—such as physical therapies, oral medications, physiokinesitherapy, or total knee arthroplasty.

### 2.2. Data Extraction

After removing duplicates and applying the “HUMAN” filter, two reviewers independently examined all of the articles for eligibility. In cases of disagreement, consultation with a third reviewer allowed for a consensus to be reached. Three reviewers independently extracted data from the included studies using a customized spreadsheet in Microsoft Excel 16.59. The following data were extracted and can be found in Table 2: (1) first author, (2) study type, (3) nationality, (4) study group population, average age, gender, radiographic grade of OA, BMI, (5) control group population, average age, gender, radiographic grade of OA, BMI, (6) type of hyaluronic acid used, (7) dosage and dosing regimen of the HA treatment program, (8) comparative treatment program, (9) measured outcomes, such as assessment scales (VAS, WOMAC, LEQUESNE, KSS, KOOS, KOS, SF-36, WHF score), gait parameters (step cadence, velocity, step length, double limb support time, sagittal ground reaction force, vertical force area, peak hip adduction moment, maximum hip flexion, hip flexion moment, hip adduction moment, peak knee valgus-varus moment, peak knee flexion-extension moment, knee abduction moment, knee adduction moment, knee flexion-extension moment), and electromyographic data.

### 2.3. Quality Assessment

**Figure 1:**
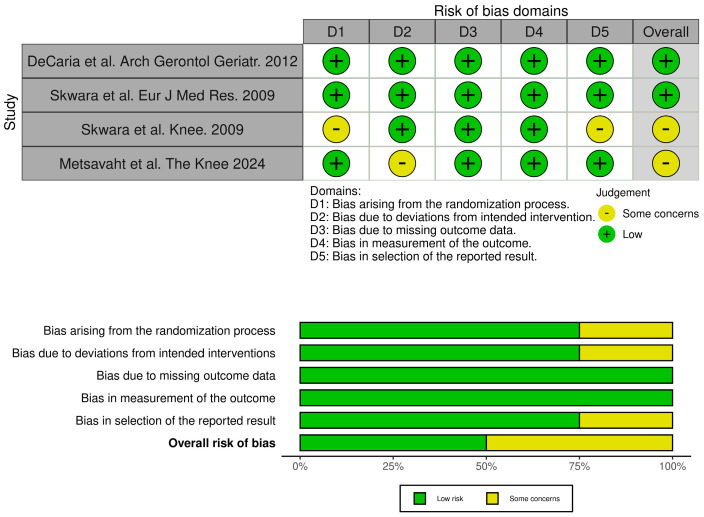
Risk assessment for RCT studies (ROB 2) [29,31,34,36,42].

**Figure 2:**
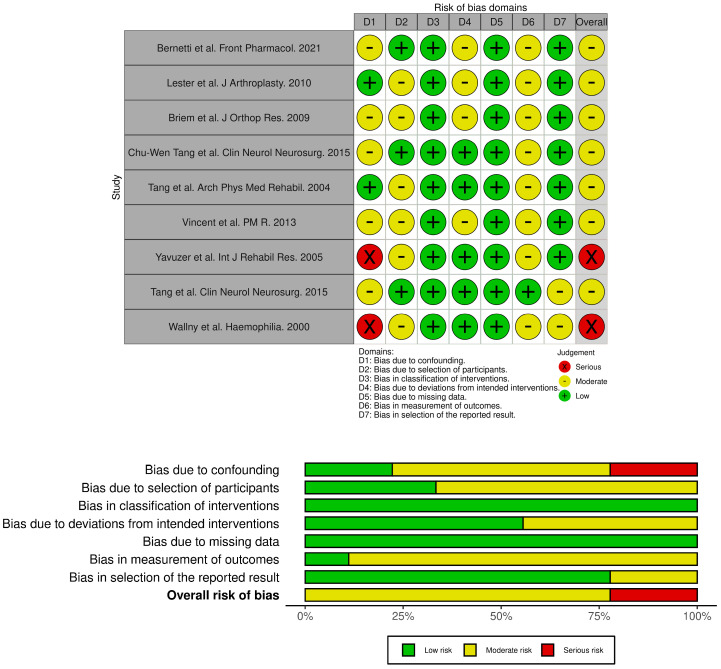
risk assessment for not randomized studies (ROBINS-I) [29,30,32,33,35,37,38,39,40,41].

The selected studies were synthesized by describing the extracted data. The quality of the articles was independently assessed by two reviewers. The quality of studies was evaluated using the RoB 2 tool for randomized controlled trials and the ROBINS-I tool for non-randomized interventional studies. Risk-of-bias judgments for each included study were graphically presented using Robvis 0.3.0 (Figure 1 and Figure 2), a web-based tool that supports the visualization of methodological quality assessments based on the ROBINS-I and RoB 2.0 frameworks [29].

**Table 2:** Main Characteristics of Studies Included.

Article	Study Design	Nationality	Study Group	Control Group	Type of HA	Intervention	Comparison	Outcomes
Bernetti et al. Front Pharmacol. 2021 [30]	QUASI-EXPERIMENTAL	Italy	n = 31; 8 M/23 F, professional or regular player (at least 2–3 times per week) KOA grade I-III	None	Hymovis MO.RE	One shot	with the baseline	WOMAC A-C KOOS VAS GAIT data collection using the ELITE system
Skwara et al. Knee. 2009 [31]	RCT	Germany	n = 21; 8 M/13 F KOA grade II–III	n = 21; 9 M/12 F grade II–III 15 participants included in the final analysis.	Linear, medium-to-high	once a week for 5 weeks	Triamcinolone acetonide	KSS Lequesne Score, VAS SF-36 Gait analysis and EMG (Helen–Hayes marker set)
Lester et al. J.Arthroplasty. 2010 [32]	QUASI-EXPERIMENTAL	USA	n = 53; 25 M/28 F KOA grade III–IV	none	Supartz High weight	once a week for 5 weeks	with the baseline	Gait analysis with Intelligent Device for Energy Expenditure and Activity (IDEEA; MiniSun, Fresno, CA)
Briem et al. J Orthop Res. 2009 [33]	QUASI-EXPERIMENTAL	Iceland	n = 27; 17 M/10 F, KOA grade I–IV	none	Hyalgan	once a week for 5 weeks	with the baseline	KOS ADL KOOS Gait analysis with Eight camera system (VICON) and two Bertec force plates
DeCaria et al. Arch Gerontol Geriatr. 2012 [34]	RCT	Canada	n = 15; 8 M/7 F; KOA grade II–III	n = 15; 8 M/7 F; KOA grade II–III	Medium-to-high	Once a week for 3 weeks + Home exercises	Placebo + Home exercises	WOMAC LK3.1 Gait analysis with GAITRite system.
Chu-Wen Tang et al. Clin Neurol Neurosurg. 2015 [35]	QUASI-EXPERIMENTAL	Taiwan	n = 23; 7 M/16 F; KOA I–II	n = 14; 5M/9F non-affected	Low-to-medium (ARTZ)	one a week for 5 weeks (bilateral)	not affected patients	Analysis of gait cycle by Vicon 370 and two AMTI force plates EMG
Skwara et al. Eur J Med Res. 2009 [36]	RCT	Germany	n = 24; 12 M/12 F; grade II–III KOA	n = 30; 15M/15F; grade II–III KOA	Durolane Cross-linked High weight	One shot	Triamcinolone acetonide	VAS Lequesne score KSS Gait analysis by Helen–Hayes markers EMG
Tang et al. Arch Phys Med Rehabil. 2004 [37]	QUASI-EXPERIMENTAL	Taiwan	n = 15, only women grade I–II KOA	n = 15, only women. non-affected	Medium weight	one per week for 5 weeks (bilateral)	not affected patients	Gait patterns with a Vicon 370 optoelectronic motion analysis system and 2 AMTI force plates
Vincent et al. PM R. 2013 [38]	Prospective cohort	USA	n = 34, 11 M/23 D grade II KOA	n = 19, 7 M/12 D grade II KOA	Medium-to-high	one a week for 3 weeks	not injected patients	NRS WOMAC SF-36 Gait analysis on 8 meter walkway
Yavuzer et al. Int J Rehabil Res. 2005 [39]	QUASI-EXPERIMENTAL	Turkyie	n = 12, 4 M/8 F KOA grade II–III.	none	Hylan G-F 20	once a week for 3 weeks (bilateral)	with the baseline	WOMAC pain, stiffness, physical functioning subscores Gait analysis with Vicon 370 system and two Bertec force plates.
Chu-Wen Tang et al. Clin Neurol Neurosurg. 2015 [40]	QUASI-EXPERIMENTAL	Taiwan	n = 25, 9 M/16 F KOA grade I–II	n = 15, 5 M/10 F healthy subjects	Medium weight (Artz)	once a week for 5 weeks (bilateral)	not affected patients	VAS Lequesne score Gait analysis with Vicon 370 and two AMTI force plates
Wallny et al. Haemophilia. 2000 [41]	PROSPECTIVE OBSERVATIONAL CASE SERIES	Germany	n = 20; hemophilic arthropathy of the knee	none	Hyalart^®^	once a week for 5 weeks	with the baseline	WFH advisory committee score Aichroth score VAS RX and MRI Biomechanical motion analysis by ultrasound topometry
Metsavaht et al. The Knee 2024 [42]	RCT	Brazil	n = 21; 5 M/16 F; KOA grade III–IV	n = 21; 5 M/16 F; KOA grade III–IV	Synolis (HA + sorbitol)	one-shot	Placebo	Gait analysis VICON motion analysis

KOA: knee osteoarthritis; KSS: Knee Society Score; ADL: activity of daily living; KOOS: Knee injury and Osteoarthritis Outcome Score; WOMAC: Western Ontario and McMaster Universities Osteoarthritis; VAS: visual analog scale; KOS: Knee Outcome Survey; PKF: peak knee flexion; PKAM1: first knee adduction moment; HA: hyaluronic acid; NRS: numerical rating scale.

## 3. Results

### 3.1. Descriptive Analysis

A total of 342 articles were found in all searches of databases. After removing duplicates, 281 papers were reviewed in terms of title and abstract, and 257 articles were excluded.

**Figure 3:**
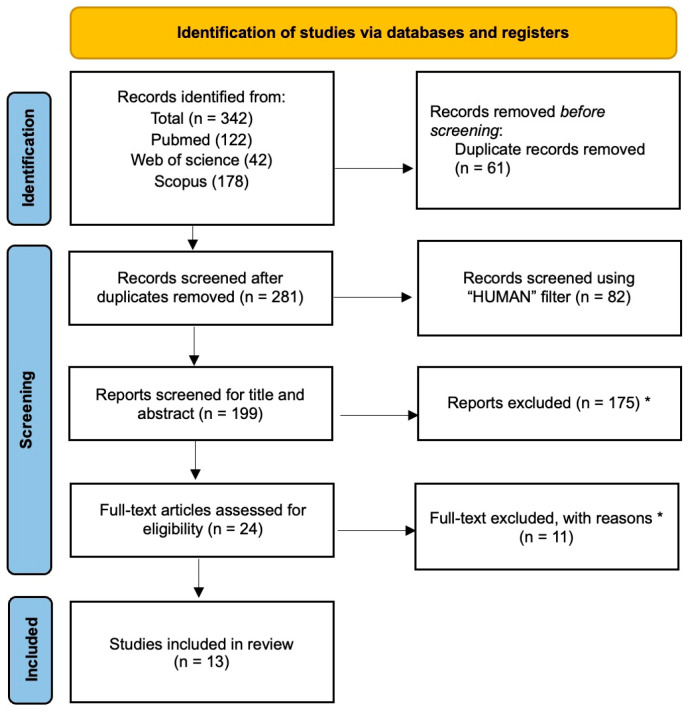
PRISMA flowchart [43]. * For details regarding the reasons for exclusion, please refer to the exclusion criteria outlined in Section 2.

Thus, we identified 24 full-text articles and retrieved them for a detailed evaluation (see Figure 3 for further details). Finally, 13 articles were included in our systematic review, of which four were RCT.

The included studies were published from 2000 to 2024. Two were conducted in Germany [31,36], two in the United States (Florida and New York) [32,38], one was conducted in Italy [30], Iceland [33], Turkey [39], Brazil [42], and three in Taiwan [35,37] [40].

Five articles compared outcomes with pre-treatment parameters of the same population, without a comparative group of study. Two articles compared the outcomes with a control group treated with intra-articular corticosteroid injections. Three articles included a control group consisting of healthy patients of the same age and sex, while one study had a control group of patients with the disease who did not receive intra-articular HA injections as a treatment for KOA. Finally, two studies included as a control group patients with a risk of KOA given intra-articular injections of a placebo.

Only one article considered patients with hemophilic arthropathy as the study population and intervention group. The remaining studies excluded patients with other comorbidities.

For gait analysis, ten studies used optoelectronic systems equipped with reflective markers, infrared cameras, and force platforms (Elite System, Vicon 370, IDEEA Minsun), two used inertial sensors (Gaitre system), and one used motion analysis ultrasound topometry.

Four articles were randomized controlled trials [31,34,36,42], one was a prospective cohort study [38], one was a prospective observational case series study [41], and the others were categorized as quasi-experimental studies [30,32,33,35,37,39,40,42].

Four hundred and ninety-one (433) patients with OA were initially selected, of whom four hundred three (403) completed the studies.

Patients studied were men and women diagnosed with grade I-IV KOA advised not to use FANS drugs during the measurements.

### 3.2. Primary Outcomes

We aimed to investigate the effects of intra-articular HA injection on kinematics, kinetics, and muscle activation patterns during walking in people with knee OA.

#### 3.2.1. Gait Parameters

In particular, we have analyzed the changes in gait patterns, distinguishing them into spatiotemporal parameters and kinematic-cinematic parameters. These parameters have been summarized in the Table 3 and Table 4.

**SPATIOTEMPORAL PARAMETERS**

*CADENCE*

Three studies evaluated cadence, i.e., the number of steps taken per minute (steps/minute). This parameter is related to the morbidity and mortality index [44].

Bernetti and colleagues [30], in their study involving individuals with an active lifestyle as professional or regular sport players affected by overuse-related knee osteoarthritis, found that cadence increased from the mean baseline value of 103.39 ± 9.07 to 110.95 ± 11.29 steps/min (*p* = 0.005) at all follow-ups. Similarly, in the study by Vincent et al. [38], cadence improved after HA injection from 97.9 ± 14.8 at baseline to 106.7 ± 10.7 steps/min after 6 months (*p* < 0.05). Tang and colleagues [40] also found a significant increase in cadence at 1 week, three months, and 6 months after HA injection (*p* < 0.05).

*WALKING SPEED*

Walking speed of distances less than 10 m is a very strong predictor of adverse health events and premature mortality.

Five of the selected studies reported a statistically significant improvement in velocity following HA injection treatment [30,31,34,38,40]. In the study by Bernetti and colleagues [30], velocity increased from a baseline value of 1.10 ± 0.14 to 1.28 ± 0.21 (*p* = 0.001). Tang AC et al. [40] also observed a significant increase in velocity at every evaluation visit (*p* < 0.001). Although DeCaria and colleagues [34] did not find a substantial difference in velocity between the HA-treated group and the placebo group, an increase in velocity greater than 10 m/sec six months after HA treatment is considered functionally significant for elderly patients with OA. Only one study [32] did not report statistically significant changes in velocity.

*STEP LENGTH AND STRIDE LENGHT*

Step length (the distance covered in a single step) and stride length (the distance between two consecutive heel strikes of the same foot) showed significant improvements in two of the selected studies. In Vincent et al. [38], step length increased from 58.6 ± 13.4 cm at baseline to 60.5 ± 13.1 cm at six months, while stride length improved from 118.0 ± 27.0 cm to 122.3 ± 26.2 cm over the same period (all *p* < 0.05). Similarly, Tang et al. [40] reported that patients with knee osteoarthritis exhibited a significantly slower walking speed (49.4% ± 11.7% of body height per second, *p* < 0.001) and shorter step length (30.8 ± 5.2% of body height, *p* = 0.01) at baseline, both of which improved following completion of intra-articular hyaluronic acid (IAHA) injections.

*SINGLE LEG STANCE*

In the prospective cohort study conducted by Vincent et al. [38], gait parameters at a self-selected walking pace were assessed using three functional tests: chair rising, stair climbing, and a 6-min walk. Notably, six-month changes in percentage of gait cycle spent in single support were all improved after HA injection (all *p* < 0.05).

Also, the HA group in the study of Metsavaht and colleagues [42] showed a reduction in single leg stance time at 1 week [−1.9%, 95% CI (−0.5 to 3.2)] and an increased total stance phase time at 12 weeks compared with the placebo group [4.3%, 95% CI (1.0 to 7.6)].

These findings can lead to a better load distribution on knee joints [42].

**KINETIC AND KINEMATIC PARAMETERS**

There are considerable variety and heterogeneity in the kinetic and kinematic parameters of gait among the selected studies. The most frequently reported improvements are observed in unilateral knee flexion excursion and knee adduction moment.

*SAGITTAL GROUND REACTION*

In the study conducted by Tang et al. [37], gait patterns and sagittal ground reaction forces (GRF) were examined in subjects with knee OA following five weekly injections of HA-IA one week after injections and over 6 months later. They reported an improvement in the characteristic 2-peak (M-shaped) force vector plot.

Skwara et al. [31] also found significant differences in the HA group for Ground Reaction Forces between the two groups at follow-up (Vertical force maximum 1, *p* = 0.018; Vertical force maximum 2, *p* = 0.019).

*KNEE FLEXION*

In the study by Chu-Wen Tang at al. [40] there were larger knee flexion moments at terminal stance and lower knee extensions among baseline and after IAHA injections in knee OA group (*p* < 0.01).

Metsavaht and colleagues [42] found differences on the sagittal plane, in particular the HA group showed increases in maximum knee extension [3.2, 95% CI (0.7 to 5.7)] and a decrease in maximum knee flexion [−3.6°, 95% CI (−6.1 to 1.2)] when compared with the placebo group, resembling a non-arthritic pattern. At 6 weeks, the HA group presented reduced maximum flexion during gait than the placebo group [−2.6°, 95% CI (−5.2 to 0.0)], but this difference was not maintained at the 12-week follow up.

Bernetti et al. [30] found statistical significance between the groups’ differences (*p* < 0.05) when comparing patients treated with HA versus controls for peak knee flexion-extension moment (treated 0.300 ± 0.242 at baseline, 0.160 ± 0.360 at 90 days of follow up, 0.200 ± 0.458 at 180 days of follow up, not treated 0.440 ± 0.340 at baseline, −0.060 ± 0.426 at 90 days of follow up, −0.110 ± 0.460 at 180 days of follow up).

*KNEE ADDUCTION MOMENT and KNEE VARO-VALGUS MOMENT*

Briem at al. [33] found the most evident changes after the HA injections in the frontal plane: the responders had an increase in the peek knee adduction moment and hip moments and kinematics contributed to increase the knee adduction moment. They found a strong correlation between pain relief and a significant increment in knee and hip moments at the frontal plane and an increase in medial co-contraction.

Bernetti and colleagues [30] found statistically significant differences between groups (*p* < 0.05) when comparing patients treated with HA versus controls for peak knee valgus varus moment (treated 0.350 ± 0.246 at baseline, 0.080 ± 0.267 at 90 days of follow up, not treated 0.430 ± 0.290 at baseline, −0.070 ± 0.285 at 90 days of follow up).

In the study of Chu-Wen Tang at al. [40], at 1 week and 3 months after IAHA injections, the knee adduction angles increased (more varus) during the initial and terminal phases of the gait cycle as compared with the knee adduction angles before IAHA injections and the control group.

In contrast to the findings of previous authors’ studies, Skwara et al. [31], showed significant differences only for knee abduction moment (*p* = 0.007) in the hyaluronan group.

*HIP KINEMATICS PARAMETERS*

In Briem’s analysis [33], at baseline, the hip of the affected limb showed significantly reduced flexion angles at PKF compared to the contralateral limb. After the hyaluronic acid injection (post-HA), the affected hip showed improvement, with greater flexion angles compared to baseline (*p* = 0.018). However, at follow-up, the affected hip still had reduced flexion angles compared to the contralateral limb, although the difference was less pronounced than before the treatment (*p* = 0.003). At follow-up, the difference between the two limbs was related to a more flexed hip at the initial contact (IC) for the affected limb. This suggests that while the PKF flexion angle improved after the treatment, there were still significant differences in the hip position at the beginning of the step.

In the same way as for the knee, Bernetti et al. [30] found statistically significant differences between groups (*p* < 0.05) when comparing patients treated with HA versus controls for peak hip ab-adduction moment and peak hip rotation moment.

For Chu-Wen Tang at al. [40], there were significant differences in the adduction moments of the patients’ hips, including larger hip adduction moments at early stance (*p* < 0.05).

Maximum hip flexion (*p* = 0.0177) and hip range of motion (*p* = 0.0043) showed a significant improvement in the two follow-ups in Skwara’s analysis [36].

**OTHER PARAMETERS**

Lester and colleagues [32] reported no significant improvements in single limb support time, walking speed, fatigue, or swing phase. However, bipedal support time increased significantly by 5.6%, from 159.74 ms before treatment to 168.65 ms after treatment (*p* = 0.04). Bernetti et al. [30] extended their analysis beyond kinetic and spatiotemporal parameters to explore correlations between clinical outcomes and biochemical measures. In the “Knee treated” group, significant correlations were observed between clinical outcomes and gait parameters. There was a negative correlation between the First Flexor-Acceptance Peak and VAS (β = −0.905, *p* = 0.011), and positive correlations between Max Knee Valgus-Varus and VAS (β = 0.717), Max Knee Valgus-Varus and KOOS-SPORT (β = 0.223, *p* = 0.032), and Range of Motion in Knee Rotation and WOMAC-A Pain, with a positive β coefficient. In contrast, Yavuzer et al. [39] observed an increase in knee joint rotation angle during walking, accompanied by reductions in extensor and adductor moments, findings that differ from those reported by other authors. Wallny and colleagues [41] studied 20 patients with hemophilic knee arthropathy and found no increase in the maximum range of motion. While 12 of the 20 patients showed improvement in biomechanical measures of rolling and sliding behavior, no significant changes were detected via MRI.

Skwara et al. [36] compared the effects of intra-articular hyaluronic acid (HA) to intra-articular triamcinolone. The gait analysis, electromyographic examination, and clinical scores showed no significant differences between the two groups. Nevertheless, both groups exhibited significant improvements in several functional parameters and pain levels.

#### 3.2.2. EMGs

Skwara et al. [31] analyzed electromyographic (EMG) activity patterns of knee-stabilizing thigh and lower leg muscles during walking. Their results showed no significant differences between the treatment and control groups in muscle activation.

Tang et al. [35] investigated changes in lower limb muscle activation following intra-articular hyaluronic acid (HA) injections. Specifically, they evaluated the EMG activity of the quadriceps (QUA), hamstrings (HAM), tibialis anterior (TA), and medial gastrocnemius (MG) muscles. During the stance phase of the gait cycle, patients with knee osteoarthritis (KOA) demonstrated prolonged muscle contraction durations in the QUA, HAM, and TA muscles compared to healthy controls. After receiving IA HA injections, muscle activity patterns in all four muscles (QUA, HAM, TA, and MG) closely resembled those of the control group. Notably, MG muscle activity began earlier during the stance phase in KOA patients prior to treatment but normalized following the completion of the injections. Furthermore, the quadriceps-to-hamstrings (Q:H) activation ratio significantly improved after IA HA administration and remained elevated for up to six months (*p* < 0.01).

### 3.3. Secondary Outcomes

The secondary outcomes examined in most of the studies included were pain, stiffness, and physical function. Pain is the most important symptom complained of by patients affected by KOA, especially during daily life. Pain during walking and painful limitation of full flexion are the most common signs [45,46]. These parameters have been summarized in the Table 5.

They were measured with several indices, including WOMAC, as in Yavuzer et al. [39], with an improvement at the follow-up (WOMAC before HA 9.2 ± 2.7 vs. 4.8 ± 3.1 after treatment, *p* = 0.005).

In five of the studies examined, knee pain during walking was assessed using VAS, and IAHA injections were shown to significantly improve the score (*p* < 0.005) from the first week after treatment [31]. In particular, the study conducted by DeCaria and colleagues demonstrated that the effect of HA on pain reduction in elderly patients with KOA is significantly greater than placebo. Bernetti at al. [30] found that VAS scores decreased significantly at all time points compared to baseline (*p* < 0.001). Even the WOMAC-A pain score and KOOS subscores decreased significantly at all time points compared to baseline (*p* < 0.001). ADL also improved (*p* < 0.001).

In the study by Skwara et al. [31], the authors evaluated the clinical efficacy of HA after a single injection compared to triamcinolone. The VAS for pain decreased in the HA group from 53.1 mm in the screening visit to 23.2 mm in the post-treatment visit and increased to 33.6 mm in the follow-up visit. There was a significant pain improvement from the screening visit to the post-treatment visit (pb0.001) and to the follow-up visit (*p* = 0.001). The LI, the Knee Society Score, and the SF-36 health questionnaire for quality of life showed a similar trend. This study highlighted a sufficient and significant pain reduction in the VAS and an improvement in Lequesne and KSS scores, as well as for triamcinolone, with the difference that the effect of HA is more long-lasting (up to 12 weeks).

Chu Weg Tang et al. [40] found the average scores of VAS and LI significantly improved after IAHA injections (*p* < 0.001) in patients with knee OA. VAS scores reduced from 54.6 ± 12.4 at baseline to 38.5 ± 11.2 at 1 week and 42.4 ± 10.0 at 6 months after IAHA injections. LI (from 14.8 ± 3.6 at baseline, to 7.4 ± 3.0 at 1 week and 8.7 ± 3.0 at 6 months after IAHA injections), pain, maximum distance walked, and difficulties in daily life scores were all significantly improved after IAHA injections from 1 week to 6 months (*p* < 0.001).

Evaluation of clinical outcomes was also performed in the study of Briem at al. [33] where KOS scores were significantly higher 3 weeks following the last injection (post-HA) (*p* = 0.005) and 5 months after treatment ended (follow-up) (*p* = 0.015). KOOS pain scores had also improved post-HA (*p* = 0.001), but less so at follow-up (*p* = 0.053).

Wallny et al. [41] evaluated the efficacy of IAHA in hemophilic patients. The average WFH score improved from 8.2 to 7, the Aichroth score increased from 38 to 40, and the VAS for subjective experience of pain fell from 5.4 to 3.8 cm. 

**Figure 4:**
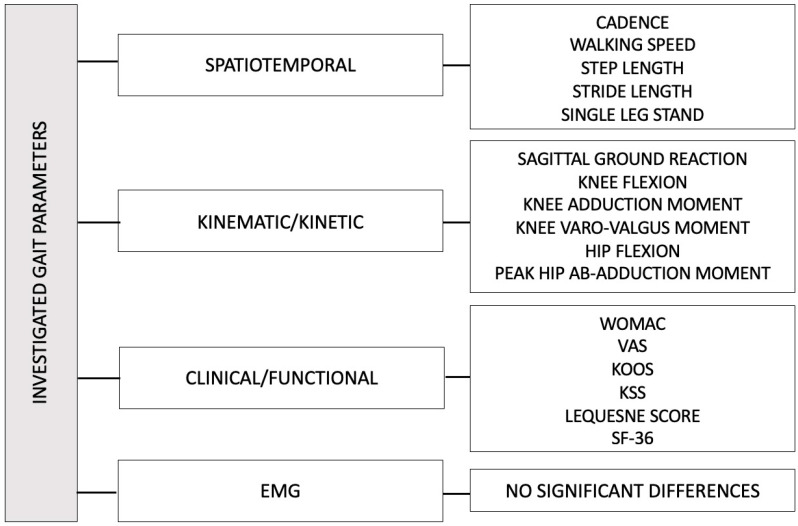
Statistically significant changes in gait parameters observed in the studies.

All key parameters that showed statistically significant changes are schematically shown in the Figure 4.

## 4. Discussion

The aim of this review was to investigate the therapeutic effect of intra-articular injections of HA in KOA, not only in terms of improving patients’ quality of life and pain but especially in terms of biomechanics and functional improvement. For this purpose, we included only articles that utilized gait analysis, which presents an objective and quantifiable instrument to detect the modifications in gait pattern occurring in patients with KOA treated with HA [47]. In literature, several authors have emphasized how gait analysis is a viable assessment tool used not only in sports science or basic biomechanical research but also as a valuable instrument in clinical practice, monitoring functional recovery during rehabilitation [13,48,49,50]. Among the gait parameters, stance phase, swing phase, single and total double support, stride length, step length, speed, and cadence are the most closely related to a physiological functional state and clinically indicative. A worsening of these parameters due to KOA may be correlated with a higher risk of falls in elderly patients or with the onset of structural or functional musculoskeletal disorders [14,51,52,53,54].

The intra-articular injection of HA in patients with KOA is a mainstay of nonoperative treatment. It leads to a significant reduction in nociceptive stimuli, as measured through VAS, WOMAC, and KOOS scales, along with an improvement in patient functionality and quality of life (ADL and qADL) [30,31,32,33,34,36,38,39,40,41], as already confirmed by numerous studies and reviews in the literature [26]. Chavda et al. [22] showed, using assessment scales as primary outcomes, that knee pain and functionality improve, even though for a limited time (usually six months). Therefore, we aimed to further investigate the effect of HA incorporating kinematic analysis, recognizing that pain is a subjective symptom.

In patients with KOA, the gait pattern is characterized by lower speed, decreased peaks of knee flexion-extension, and reduced loading [39]. These alterations are chronically established and are difficult to address.

In general, despite the heterogeneity of protocols and types of HA used, it emerges that in patients with moderate KOA and modifications in walking parameters, intra-articular injections of HA resulted in statistically significant improvements of gait. In particular, the predominant role of viscosupplementation in responder patients is evident in terms of increased self-selected speed, cadence, and peak knee flexion-extension [30,40,42]. Moreover, some studies have also highlighted an increase in knee adduction moment, a parameter related to increased walking speed. Chu-Wen Tang and colleagues reported that the pain scores exhibited a negative correlation with knee adduction moments at early stance from baseline to post-IAHA injections. The reduction in pain appears to be directly proportional to the increase in knee adduction moment [40].

On the other hand, some gait parameters appear not to show clear post-injection improvements. Although numerous studies support the clinical and biomechanical benefits of intra-articular hyaluronic acid (HA) injections in the management of knee osteoarthritis, some trials have not demonstrated statistically significant improvements. This variability may be attributed to several factors. First, the placebo effect associated with intra-articular procedures can be substantial, particularly for subjective outcomes such as pain, potentially obscuring the true therapeutic efficacy of HA, especially in studies without a control group. Second, there is considerable heterogeneity in the types and formulations of HA used across studies. These include variations in molecular weight (high versus low), structure (cross-linked versus non-cross-linked), and administration protocols such as single versus multiple injections. These differences can influence both the extent and duration of the clinical response. Third, patient-specific factors likely contribute to treatment variability. These include disease severity, joint alignment, physical activity level (for example, athletes compared to older adults), and the presence of comorbidities. For instance, trials involving athletic populations [30] may yield different outcomes compared to those focusing on elderly or hemophilic patients [41]. This heterogeneity highlights the importance of patient stratification and personalized treatment strategies when evaluating the efficacy of HA injections for knee osteoarthritis.

Furthermore, authors emphasized how the increase of knee adduction moment, along with a decrease in pain, could be disadvantageous for the joints of patients with medial knee osteoarthritis [30,33,40]. It can lead to an accelerated rate of degeneration during frequent functional activities like walking. It has been demonstrated that a 1% increase in the knee adduction moment can elevate the risk of degenerative progression by 6.46 times [55].

These results align with findings from Henriksen et al., stating that when a treatment reduces pain, the patient is less likely to unload the affected compartment. An increase in co-contraction in association with pain reduction may reflect an unsuccessful attempt to stabilize the medial compartment as it is more solicited. The combination of increased load on the medial compartment, co-contraction as an attempt to stabilize the knee, and no improvements in parameters in the frontal plane can lead to overloading a vulnerable region already affected by the osteoarthritic process [56,57,58].

Thus, considering the improvements and biomechanical modifications induced by intra-articular HA injections, as well as the parameters that do not improve or that could lead to osteoarthritis progression, some authors highlighted the importance of complementary rehabilitation to intra-articular HA injections. Joint pain leads to postural and kinematic adaptations of the gait (pain avoiding patterns). In particular, individuals with osteoarthritis exhibit altered gait kinematics due to impaired weight-bearing on the affected limb, often resulting in muscle weakness, particularly of the quadriceps [1]. This is commonly compensated for by adaptive mechanisms at the ankle and hip joints. With the reduction of the pain, due to the effect of hyaluronic acid, the patients may continue to walk with the pre-existing abnormal gait patterns. Contrary to expectations, this could exacerbate osteoarthritis by increasing the load on the already compromised joint compartment, leading to further overload of the medial component of the knee and thus worsening the condition. In this context, it may therefore be important to incorporate targeted rehabilitation based on kinematic data, in order to optimize load distribution in the frontal plane, particularly in patients with medial compartment osteoarthritis, thus maximizing the analgesic benefits of hyaluronic acid. Hip muscle reinforcement with personalized rehabilitation plans, physical therapy, visual feedback therapy, and combinations of intra-articular injections can have a synergistic effect on functional scores, preventing compensations [59,60,61].

In the randomized controlled study of Saccomanno et al., the researchers evaluated how intra-articular injections of HA and individually tailored rehabilitation programs, administered alone or in combination, are effective in improving knee function and pain relief [62]. One hundred sixty-five patients affected by moderate degrees of knee OA were randomly divided into three groups. Group 1 (HA) underwent three HA injections (one every 2 weeks); group 2 (EBR) underwent 20 treatment sessions in a month of an individualized program; and group 3 (HA + EBR) received both treatments simultaneously. The combined treatment showed the statistically significative pain relief at 1-month follow-up compared to either in isolation, in term of improvement of WOMAC pain, stiffness, and function subscales. Furthermore, in the review of Monticone and colleagues, physical therapy agents (TENS) and home exercises compared to intra-articular injection seem to be equally effective on pain, disability, and quality of life. The authors concluded suggesting a treatment model associating intra-articular viscosupplementation, physical, and rehabilitative interventions to improve functional outcomes [61].

Moreover, in particular selected cases, it is possible to associate HA with CS and PRP to achieve better results. In particular, Smith C. and colleagues demonstrated that combined injections of HA and CS allow better symptom management up to a year later than using viscosupplement therapy alone, in term of WOMAC pain, WOMAC total, and OMERACT-OARSI responder rate improvement [63]. Similarly, Karasavvidis T. and colleagues, in their meta-analysis, evaluated the combination effect of viscosupplementation therapy with HA coupled with PRP, identifying improvements lasting up to a year in the VAS and WOMAC scores [64].

In future perspectives on KOA management, research should address the effectiveness of the association of HA with the wide range of interventions in KOA patients. Many authors have primarily studied alternative treatments not in association with viscosupplementation for the treatment of knee osteoarthritis, finding satisfactory results in terms of pain and gait. As reported in the study of Fransen and colleagues, physical therapy and hip muscle strengthening represent an effective treatment method to reduce knee adduction in patients with OA monitored through gait analysis after individual or group treatment [65]. This study revealed that physical therapy, in the form of individual treatments (20 min of muscle strengthening exercise or manual techniques aimed at increasing range of motion and 5–10 min of an electrophysical agent such as heat, ultrasound, laser, or interferential therapy) or group format (1 h twice a week for 8 weeks supplemented with a home exercise program of 3 days per week stretches sessions followed by 20 min of continuous outdoor walking or indoor stationary bicycle), resulted in significantly increased isometric muscle strength, gait speed, stride length and pain, physical function, and health related quality of life above controls [65].

Alternative interventions such as the use of orthotics have demonstrated effectiveness in reducing knee adduction moments. One study has shown that a valgus-inducing knee brace could compensate for approximately 10% of adduction moment [66]. In a review by Yan et al. the valgus orthosis can be used to relieve the symptoms of patients with medial gonarthrosis by decreasing the varus angle, decreasing the knee adduction moment (KAM), and redistributing the knee compartment loads. However, the effects of valgus braces on other biomechanical parameters (e.g., walking speed, cadence, joint angle, and joint space) had not reached a consensus [59].

Moreover, Srinivasan et al., in their study involving 91 patients with symptomatic medial compartmental KOA randomized to treatment with either a 10-mm laterally wedged insole or a valgus brace, showed that a long-term application of lateral wedged insole can also induce gait adaptations, leading to reduced adduction moments when walking [67].

Based on these premises, the association of HA with exercise and orthoses could represent the right approach to improve knee function and gait in patients with KOA to overcome the limitation of the HA on walking parameters.

However, the literature lacks studies evaluating the effectiveness of complementary therapeutic programs using HA in combination with other approaches such as rehabilitation, orthotic use, or physical therapy, with gait analysis as follow-up. There is a need for more in-depth studies to assess the efficacy of such combined therapeutic programs.

Physicians should longitudinally follow patients after HA injections to plan a rehabilitation program based on the patient’s deficit. In this context, gait analysis represents a valid clinical tool, providing a deeper insight on patient biomechanics. However, the correct laboratory setting plays a crucial role in meeting the clinical or research needs. In general, optoelectronic systems, which represent the gold standard, are usually reserved for research purposes. Inertial measurement units represent a good choice in clinical settings. New technologies such as 3D camera treadmills could gain great importance during rehabilitation for both evaluation purposes and biofeedback therapy [1,13,68].

The main limitations of this literature review stem from the small and heterogeneous patient samples, which contribute to variability in the gait parameters analyzed, some of which show only minimal alterations at baseline. Moreover, due to the substantial heterogeneity among the included studies in terms of evaluated outcomes and applied protocols, a quantitative synthesis (meta-analysis) could not be performed. In addition, many studies do not employ blinding, and there is a lack of standardization regarding the molecular weight of hyaluronic acid and the injection protocols used, further limiting the comparability and applicability of the findings. Furthermore, the current literature lacks a substantial number of long-term studies with follow-up periods extending beyond six months.

## 5. Conclusions

HA injections may be associated with improvements in certain gait parameters, including walking speed, cadence, and knee joint mobility. These findings indicate a potential positive impact of HA on functional performance. Combining HA injections with targeted rehabilitation protocols could further support these improvements, although more research is needed to confirm this. Gait analysis remains a useful tool to monitor treatment response and help tailoring rehabilitation strategies.

## Figures and Tables

**Table 1:** Search strategy.

**PubMed** ((gait analysis) OR (gait) OR (walk) OR (inertial sensor) OR (inertial measurement unit) OR (accelerometer) OR (surface electromyography) OR (kinematic analysis)) AND (knee) AND ((arthrosis) OR (arthritis) OR (osteoarthrosis) OR (gonarthritis) OR (gonarthrosis)) AND ((injection) OR (infiltration)) AND ((viscosupplementation) OR (viscoinduction) OR (hyaluronic acid))
**Web of Science** (((gait analysis) OR (gait) OR (walk) OR (inertial sensor) OR (inertial measurement unit) OR (accelerometer) OR (surface electromyography) OR (kinematic analysis)) AND (knee) AND ((arthrosis) OR (arthritis) OR (osteoarthrosis) OR (gonarthritis) OR (gonarthrosis)) AND ((injection) OR (infiltration)) AND ((viscosupplementation) OR (viscoinduction) OR (hyaluronic acid)))
**Scopus** TITLE-ABS-KEY((“gait analysis”) OR (“gait”) OR (“walk”) OR (“inertial sensor”) OR (“inertial measurement unit”) OR (“accelerometer”) OR (“surface electromyography”) OR (“kinematic analysis”)) AND (“knee”) AND ((“arthrosis”) OR (“arthritis”) OR (“osteoarthrosis”) OR (“gonarthritis”) OR (“gonarthrosis”)) AND ((“injection”) OR (“infiltration”)) AND ((“viscosupplementation”) OR (“viscoinduction”) OR (“hyaluronic acid”))

**Table 3:** Summary of statistically significant changes in spatiotemporal parameters.

Variables	Study	Follow up Time	Intervention Mean	Comparison Mean	*p* Value
**CADENCE** (step/minute)	Bernetti et al., 2021 [30]	Baseline	103.39 ± 9.07	/	*p* = 0.005
		Day 30	/		
		Day 90	110.95 ± 11.29		
		Day 180	/		
		Day 360	/		
	Vincent et al., 2013 [38]	Baseline	97.9 ± 14.8	108.1 ± 10.4	*p* < 0.05
		6 months	106.7 ± 10.7	108.6 ± 10.8	
	Tang et al., 2004 [37]	Baseline	96.1 ± 11.0	106.3 ± 12.3	*p* < 0.05
		1 week	108.8 ± 10.1		
		3 months	110.7 ± 12.01		
		6 months	12.7 ± 14.3		
**WALKING SPEED** m/s	Bernetti et al., 2021 [30]	Baseline	1.10 ± 0.14	/	*p* = 0.001
		Day 30	/		
		Day 90	1.28 ± 0.21		
		Day 180	/		
		Day 360	/		
	Vincent et al., 2013 (cm/s) [38]	Baseline	98.0 ± 31.4	118.8 ± 22.9	*p* = 0.002
		6 months	110.2 ± 30.4	118.6 ± 23.1	
	Tang AC et al., 2015 (%BH/sec) [40]	Baseline	49.4% ± 11.7	~62(box plot)	*p* < 0.001
		1 week	~58(box plot)		
		3 months	~60(box plot)		
		6 months	~60(box plot)		
	DeCaria et al., 2012 [34]	Week 4 3 months 6 months	12.32 (11.50) 11.10 (11.43) 9.53 (14.33)	8.23 (10.46) 9.86 (10.22) 12.83 (11.89)	*p* = 0.01
	Tang et al., 2004 (%BH/sec) [37]	Baseline	50.2 ± 11.9	59.5 ± 10.7	*p* < 0.05
		1 week	62.2 ± 10.8		
		3 months	63.1 ± 16.8		
		6 months	61.8 ± 16.2		
**STEP LENGTH** cm	Vincent et al., 2013 [38]	Baseline	58.6 ± 13.4	67.4 ± 15.5	*p* < 0.05
		6 month	60.5 ± 13.1	66.9 ± 15.8	
	Tang AC et al., 2015 [40]	Baseline	30.8 ± 5.2	~38(box plot)	*p* = 0.01
		1 week	~35(box plot)		
		3 months	~34(box plot)		
		6 months	~33(box plot)		
	Tang et al., 2004 [37]	Baseline	30.4± 5.4	35.6 ± 4.3	*p* < 0.05
		1 week	34.6 ± 4.7		
		3 months	33.7 ± 7.0		
		6 months	33.5 ± 6.8		
**STRIDE LENGTH** cm	Vincent et al., 2013 [38]	Baseline	118.0 ± 27.0	131.4 ± 18.0 126.6 ± 29.5	*p* < 0.05
		6 month	122.34 ± 26.2		
**SINGLE LEG STANCE** sec	Vincent et al., 2013 [38]	Baseline	32.8 ± 4.0	35.4 ± 2.1	*p* < 0.05
		6 month	33.8 ± 4.2	35.3 ± 1.9	
	Metsavaht et al., 2024 [42]	Baseline	38.8%	38.0%	*p* < 0.05
		1 week	38.4%	39.6%	
		6 weeks	38.8 %	38.5 %	
		8 weeks	38.3%	38.9%	
**BIPEDAL SUPPORT TIME** (ms)	Lester et al., 2010 [32]	Baseline 3 weeks	159.74	168.65	*p* = 0.04

m/s: meters per second; cm/s: centimeters per second; %BH/sec: percent of body height per second.

**Table 4:** Summary of statistically significant changes in Kinetic and Kinematic parameters.

Variables	Study	Follow Up Time	Intervention Mean	Comparison Mean	*p* Value
**SAGITTAL GROUND REACTION** 1st peak force (%BW)	Tang et al., 2004 [37]	Baseline	100.7 ± 5.1	102.3 ± 6.5	*p* < 0.05
		1 week	103.5 ± 10.0		
		3 months	106.0 ± 8.7		
		6 months	105.5 ± 6.9		
	Skwara et al., 2009 [31] *Vertical force maximum 1 and 2 (BW)*	Baseline 1 week 12 weeks	1.1 / 1.1	1.0 / 1.0	*p* = 0.018
**KNEE FLEXION** (Nm/Kg)	Tang AC et al., 2015 [40]	Baseline 1 week 3 months 6 months	larger knee flexion moments at terminal stance (graphical data)	/	*p* < 0.01
	Metsavaht et al., 2024 (angle°) [42]	baseline	12.9	14.8	*p* < 0.001
		1 week	11.0	14.8	
		6 week	12.7	15.5	
		12 week	13.1	14.2	
	Bernetti et al., 2021 [30]	Baseline	0.300 ± 0.242	0.440 ± 0.340	*p* < 0.05
		Day 30	0.080 ± 0.380	0.540 ± 2.377	
		Day 90	0.160 ± 0.360	−0.06 ± 0.426	
		Day 180	0.200 ± 0.458	−0.110 ± 0.460	
		Day 360	0.190 ± 0.970	−0.10 ± 0.399	
**KNEE ADDUCTION MOMENT and KNEE VARO-VALGUS MOMENT Nm/Kg**	Briem et al., 2009 [33]	Baseline 3 weeks 5 months	Increased peek knee adduction moment (graphical data)	/	*p* = 0.001
	Bernetti et al., 2021 [30]	Baseline	0.350 ± 0.246	0.430 ± 0.290	*p* < 0.05
		Day 30	0.030 ± 0.250	0.060 ± 0.341	
		Day 90	0.080 ± 0.267	−0.07 ± 0.285	
		Day 180	0.160 ± 0.269	0.050 ± 0.242	
		Day 360	0.080 ± 0.298	0.100 ± 0.360	
	Tang AC et al., 2015 [40]	Baseline	increased	/	*p* < 0.001
		1 week	(more varus)		
		3 months	(graphical data)		
		6 months			
	Yavuzer et al., 2005 [39]	Baseline	0.45 ± 0.1	/	*p* = 0.047
		1 week	0.41 ± 0.1		
**KNEE ABDUCTION MOMENT Nm/Kg**	Skwara et al., 2009 [31]	Baseline	/	/	*p* = 0.007
		1 week	0.2	0.3	
		12 weeks	0.3	0.3	
**HIP FLEXION ANGLE**	Briem et al., 2009 [33]	Baseline	Increased hip	/	*p* = 0.018
		3 weeks	flexion angle		
		5 months	(graphical data)		
	Skwara et al., 2009 [36]	Baseline	38.74	36.16	*p* = 0.0177
		12 weeks	42.48	38.33	
**PEAK HIP** **AB-ADDUCTION MOMENT**	Bernetti et al., 2021 [30]	Baseline	0.490 ± 0.296	0.560 ± 0.304	*p* < 0.05
		Day 30	0.120 ± 0.392	0.170 ± 0.471	
		Day 90	0.190 ± 0.383	0.000 ± 0.341	
		Day 180	0.220 ± 0.353	0.140 ± 0.521	
		Day 360	0.050 ± 0.398	0.070 ± 0.444	
	Tang AC et al., 2015 [40]	Baseline 1 week 3 months 6 months	Increased adduction at early stance (graphical data)	/	*p* < 0.05

**Table 5:** Summary of statistically significant changes in clinical outcomes.

Variables	Study	Follow Up Time	Intervention Mean	Comparison Mean	*p* Value
**WOMAC pain**	Yavuzer et al., 2005 [39]	Baseline	9.2 ± 2.7	/	*p* = 0.005
		1 week	4.8 ± 3.1		
	Bernetti et al., 2021 [30]	Baseline	11.77 ± 10.53	/	*p* < 0.001
		Day 30	11.77 ± 10.53		
		Day 90	7.410 ± 9.320		
		Day 180	8.040 ± 9.620		
		Day 360	8.040 ± 9.620		
	Vincent et al., 2013 [38]	Baseline	7.5 ± 3.7	5.7 ± 3.3	*p* = 0.009
		6 month	5.5 ± 4.7	7.2 ± 5.2	
	DeCaria et al., 2012 [34]	Baseline	11.25 ± 4.17		*p* = 0.008
		1 week	8.50 ± 2.98		
**VAS mm**	Skwara et al., 2009 [31]	Baseline	53.1	57.9	*p* < 0.001
		1 week	23.2	20.5	
		12 weeks	33.6	32.00	
	Bernetti et al., 2021 [30]	Baseline	68.65 ± 11.53	/	*p* < 0.001
		Day 30	20.94 ± 19.10		
		Day 90	16.48 ± 18.65		
		Day 180	12.15 ± 17.29		
		Day 360	14.39 ± 15.41		
	Tang AC et al., 2015 [40]	Baseline	54.6 ± 12.4	/	*p* < 0.001
		1 week	38.5 ± 11.2		
		3 months	36.8 ± 10.3		
		6 month	42.4 ± 10.0		
	Wallny et al., 2000 (cm) [41]	Baseline	5.4	/	/
		3 months	3.8		
**KOOS**	Bernetti et al., 2021 [30]	Baseline	41.61 ± 20.95	/	*p* < 0.001
		Day 30	74.66 ± 21.63		
		Day 90	74.66 ± 21.63		
		Day 180	76.15 ± 18.46		
		Day 360	78.04 ± 19.53		
	Briem et al., 2009 [33]	Baseline	61	61	*p* = 0.001
		3 weeks	79	61	
		5 months	75	66	
**KSS**	Skwara et al., 2009 [31]	Baseline	128.2	132.1	*p* < 0.001
		1 week	147.0	155.7	
		12 weeks	140.6	154.7	
**LI**	Skwara et al., 2009 [31]	Baseline	11.5	12.3	*p* < 0.001
		1 week	8.3	7.6	
		12 weeks	8.8	9.1	
	Tang AC et al., 2015 [40]	Baseline	5.6 ± 1.9	/	*p* = 0.01
		1 week	2.2 ± 1.0		
		3 months	2.4 ± 1.7		
		6 months	3.0 ± 1.5		
**KOS**	Briem et al., 2009 [33]	Baseline	62	71	*p* = 0.005
		3 weeks	78	73	
		5 months	76	72	
**SF36**	Skwara et al., 2009 [31,36]	Baseline 1 week 12 weeks	Improved (graphical data)	/	*p* = 0.002
					
	Vincent et al., 2013 [38]				
	*Role Physical*	Baseline	33.6 ± 10.3	49.1 ± 9.2	*p* = 0.025
		6 months	35.4 ± 10.6	37.7 ± 10.6	
	*Vitality*	Baseline	44.6 ± 8.2	50.9 ± 7.4	*p* = 0.013
		6 months	50.4 ± 5.9	48.8 ± 10.8	
	*Role Emotional*	Baseline	38.9 ± 13.8	50.0 ± 5.6	*p* = 0.024
		6 months	45.8 ± 12.7	51.3 ± 11.1	
